# Transaldolase in *Bacillus methanolicus*: biochemical characterization and biological role in ribulose monophosphate cycle

**DOI:** 10.1186/s12866-020-01750-6

**Published:** 2020-03-24

**Authors:** Johannes Pfeifenschneider, Benno Markert, Jessica Stolzenberger, Trygve Brautaset, Volker F. Wendisch

**Affiliations:** 1 Genetics of Prokaryotes, Faculty of Biology & Center for Biotechnology, Bielefeld University, Universitätsstraße 25, 33615 Bielefeld, Germany; 2 Department of Biotechnology, NTNU, Norwegian University of Science and Technology, Trondheim, Norway

**Keywords:** Methylotrophy, Transaldolase, RuMP cycle, *Bacillus methanolicus*

## Abstract

**Background:**

The Gram-positive facultative methylotrophic bacterium *Bacillus methanolicus* uses the sedoheptulose-1,7-bisphosphatase (SBPase) variant of the ribulose monophosphate (RuMP) cycle for growth on the C_1_ carbon source methanol. Previous genome sequencing of the physiologically different *B. methanolicus* wild-type strains MGA3 and PB1 has unraveled all putative RuMP cycle genes and later, several of the RuMP cycle enzymes of MGA3 have been biochemically characterized. In this study, the focus was on the characterization of the transaldolase (Ta) and its possible role in the RuMP cycle in *B. methanolicus*.

**Results:**

The Ta genes of *B. methanolicus* MGA3 and PB1 were recombinantly expressed in *Escherichia coli*, and the gene products were purified and characterized. The PB1 Ta protein was found to be active as a homodimer with a molecular weight of 54 kDa and displayed K_M_ of 0.74 mM and V_max_ of 16.3 U/mg using Fructose-6 phosphate as the substrate. In contrast, the MGA3 Ta gene, which encodes a truncated Ta protein lacking 80 amino acids at the N-terminus, showed no Ta activity. Seven different mutant genes expressing various full-length MGA3 Ta proteins were constructed and all gene products displayed Ta activities. Moreover, MGA3 cells displayed Ta activities similar as PB1 cells in crude extracts.

**Conclusions:**

While it is well established that *B. methanolicus* can use the SBPase variant of the RuMP cycle this study indicates that *B. methanolicus* possesses Ta activity and may also operate the Ta variant of the RuMP.

## Background

There are three groups of methylotrophic bacteria using the ribulose monophosphate (RuMP) cycle for the fixation of formaldehyde: Gram-negative obligate and facultative methylotrophs and Gram-positive facultative methylotrophs [3, 4]. *Bacillus methanolicus*, a facultative methylotroph, belongs to the latter group and utilizes methanol as its sole source of energy and carbon, assimilating formaldehyde via the RuMP cycle [33, 53]. *B. methanolicus* is a valuable host for industrial fermentations using methanol as sole carbon source [12, 14, 27, 44]. The oxidation of methanol to formaldehyde, the first step of methanol utilization in *B. methanolicus,* is catalyzed by NAD-dependent methanol dehydrogenase [6, 15, 24]. It has been shown that *B. methanolicus* possesses three different genes, all encoding active Mdhs [23, 36].

**Fig. 1:**
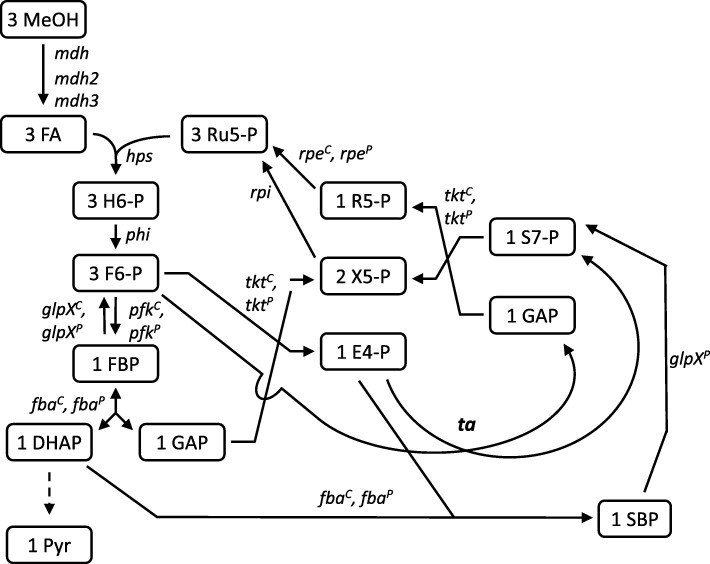
Schematic representation of the RuMP as present in *B. methanolicus*. Reactions are represented by arrows and the genes encoding the respective enzymes are depicted next to them. For abbreviations of the genes see the text. Abbreviations for metabolites are H6-P, 3-hexulose 6-phosphate; F6-P, fructose-6-phosphate; FBP, fructose-1,6-bisphosphate; GAP, glyceraldehyde 3-phosphate; DHAP, dihydroxyacetone phosphate; E4-P, erythrose 4-phosphate; SBP, sedoheptulose 1,7-bisphosphate; S7-P, sedoheptulose-7-phosphate; Ri5-P, ribose 5-phosphate; X5P, xylulose 5-phosphate; Ru5P, ribulose 5-phosphate

The RuMP cycle consists of three main parts: fixation, cleavage, and rearrangement (Fig. 1). In the fixation part, ribulose 5-phosphate (Ru5P) is condensed with formaldehyde by 3-hexulose-6-phosphate synthase (Hps) to yield hexulose 6-phosphate (H6P), which is subsequently isomerized to fructose 6-phosphate by 6-phospho-3-hexuloisomerase (Phi). Mdh, Hps, and Phi are specific enzymes of methylotrophy, whereas the other involved enzymes are also part of other pathways, such as glycolysis, Calvin cycle, and pentose phosphate pathway. In the cleavage part, F6P is converted by phosphofructokinase (Pfk) to fructose 1,6-bisphosphatase (FBP), which is further cleaved into the triose phosphates glyceraldehyde 3-phosphate (GAP) and dihydroxyacetone phosphate (DHAP) by fructose-1,6-bisphosphate aldolase (Fba).

Genome sequencing of the two physiologically different *B. methanolicus* wild-type strains MGA3 and PB1 that showed considerable differences with respect to growth, amino acid production, and respiration profiles in fed-batch methanol cultivations [23] provided genetic insight into all methylotrophic pathways of this species ([23]; Irla et al. 2014). Both strains carry the methylotrophy plasmids pBM19 and pBM20, respectively, and MGA3 in addition carry the natural and cryptic plasmid pBM69. *B. methanolicus* has two Fba enzymes, one encoded on the chromosome and one plasmid encoded variant (genes *fba*^*C*^ and *fba*^*P*^, respectively). They catalyze opposite reactions, since Fba^C^ is the major glycolytic Fba, whereas Fba^P^ catalyzes the aldol condensation in gluconeogenesis [62]. Interestingly, *B. methanolicus* possesses enzymes for the sedoheptulose-1,7-bisphosphatase (SBPase) variant as well as the transaldolase (Ta) variant of the rearrangement part of the RuMP cycle. In this part, F6P and the triosephosphates are converted into Ru5P, which is thereby regenerated for the fixation of formaldehyde. In the SBPase variant, erythrose 4-phosphate (E4P) and DHAP are condensed to sedoheptulose 1,7-bisphosphate (SBP), catalyzed by either Fba^P^ or Fba^C^. Therefore, these enzymes function also as sedoheptulose-1,7-bisphosphate aldolases [61]. In the following step, SBPase dephosphorylates SBP to yield S7P. Both the chromosomally encoded FBPase GlpX^C^ and the plasmid encoded FBPase/SBPase GlpX^P^ are active as bisphosphatases with FBP, whereas only the plasmid encoded GlpX^P^ was found to function as SBPase [61]. In the Ta variant, E4P and F6P are directly converted into GAP and S7P. Both variants of the RuMP cycle involve the enzymes transketolase (Tkt) [39], ribose-5-phosphate isomerase (Rpi), and ribulose-phosphate 3-epimerase (Rpe). Previous studies on gene regulation and extensive enzyme characterizations has concluded that *B. methanolicus* MGA3 uses the SBPase variant, and the biological significance of the annotated Ta genes is unknown.

The genes of the rearrangement part are present in two versions in *B. methanolicus*; one encoded on the chromosome and the other one on the naturally occurring plasmid pBM19. The Ta and Rpi genes are exceptional as they are only present on the chromosome [11, 23].

Ta catalyzes the transfer of a dihydroxyacetone moiety from a ketose donor onto an aldehyde acceptor. This enzyme does not require any known cofactors and performs a base-catalyzed aldol cleavage reaction via a Schiff base intermediate. Several Tas have been purified and characterized from various sources, such as bacteria [60], archaea [59], yeasts [18], fungi [35], plants [41], and mammals, including humans [9]. The only thermostable Ta characterized to date is from *Methanocaldococcus jannaschii* [59]. Five subfamilies can be distinguished, based on the phylogeny of Ta [50]. Subfamily I is considered as the classical Ta that occurs in all domains of life, for example, TalB from *E. coli* [60], but not in plants. Subfamily II and subfamily III Tas are present in plants and in the case of subfamily III also in bacteria. Subfamily IV consists of small Tas found in bacteria and archaea. To the best of our knowledge, biochemical evidence for Ta enzyme activity has not been shown yet for enzymes belonging to either subfamily II or V [51]. Interestingly, enzymes of subfamily V are active as F6P aldolases as shown for respective enzymes from *E. coli* [57]. However, certain organisms, like *Zymomobas mobilis* [17], and *Entamoeba histolytica* [65], as well as certain mammalian tissues do not express Ta [21] and the non-oxidative branch can function without this enzyme [40, 45].

In this study we investigated the biochemical properties and biological function of the putative Ta proteins in *B. methanolicus* MGA3 and PB1 [47]. The MGA3 Ta gene encodes a truncated and non-active protein (UNIPROT IDI3EBM5) while the PB1 Ta gene product (I3E1B6) displayed Ta activity, tested both in vitro and in vivo. Interestingly, genetically repaired full-length versions of the MGA3 Ta gene showed Ta activity. The PB1 enzyme was biochemically characterized and our results may indicate that *B. methanolicus* has an operative TA variant of the RuMP cycle for methylotrophic growth.

## Results

### The genomes from *B. methanolicus* PB1 and MGA3 encode transaldolase

**Fig. 2:**
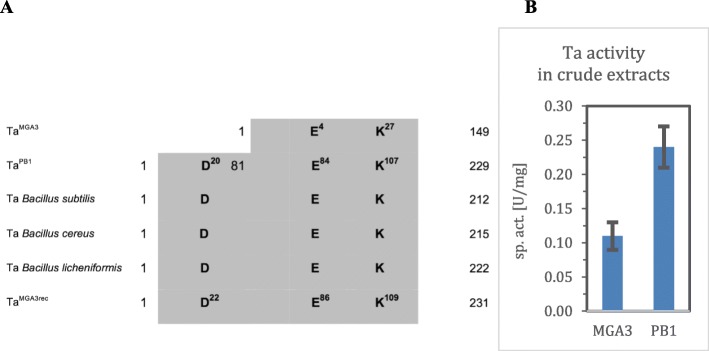
**a** Schematic comparison of transaldolases from *B. methanolicus* strains MGA3 and PB1 with transaldolases from *Bacillus subtilis, Bacillus cereus,* and *Bacillus licheniformis*. Shaded boxes represent the protein sequence, the number in front of and behind the shaded boxes represent the first and last amino acid. Residues of the catalytic active center are given in one letter code with their position in the protein in superscript. D, E, and K represent the amino acids aspartate, glutamate, and lysine. **b** Ta activities measured in crude extracts of *B. methanolicus* strains MGA3 and PB1

Inspection of the genome sequences of *B. methanolicus* PB1 and MGA3 revealed that each strain possesses a single chromosomally encoded transaldolase, while no Ta gene was found on the plasmids pBM20 and pBM19. Bioinformatics comparison of the deduced primary amino acid sequences of these Ta proteins, MGA3 (Ta^MGA3^) and PB1 (Ta^PB1^) [23, 25], with Ta proteins from other *Bacillus* species (Fig. 2a) revealed that they can be associated with group IV family of Ta enzymes. This comparison surprisingly shows that Ta^MGA3^ lacks about 80 N-terminal amino acids that are present in Ta^PB1^ and the other Tas compared here. Aspartate residue D20 is part of the catalytic triad D20-E84-K107 of Ta enzymes [50, 55] and this triad is present in Ta^PB1^, but D20 is absent from Ta^MGA3^. Thus, Ta^MGA3^ likely lacks Ta activity.

**Fig. 3:**
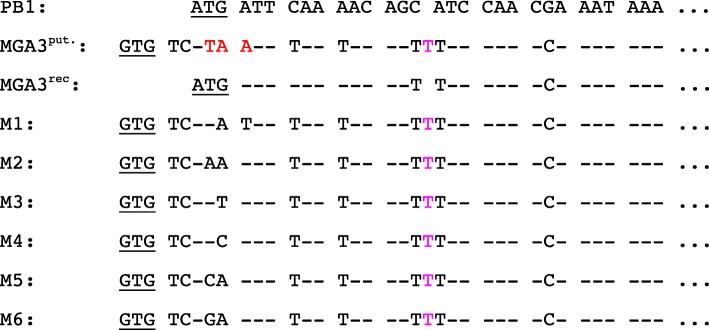
Comparison of the nucleotide sequences of the Ta genes from *B. methanolicus* PB1 and MGA3 as well as those of recreated MGA3 Ta genes. MGA3^put^ represents an alternative Ta coding sequence of MGA3. For MGA3^rec^ the ATA was changed to ATG and adjusted to that of PB1. For the variants M1-M6 the third codon of the sequence upstream of *ta*^*MGA3*^ was changed from a stop TAA codon to different codons. The dashes indicate the same nucleotide as for PB1 and the dots show that the sequence continues. The red letters indicate a stop codon and the pink letter shows an additional nucleotide at that position. A rough screen showed that crude extracts of *E. coli* BL21 (DE3) expressing all of these proteins except for Ta^MGA3^contained at least 20 mU/mg, i.e. two fold of the activity present in the parent strain (about 10 mU/mg)

Closer inspection of the gene sequences revealed several single nucleotide polymorphisms (SNPs) and suggested that the putative translation initiation codon ATG present in PB1 is mutated to ATA in MGA3 (Fig. 3). An alternative GTG start codon only leads to a peptide with two amino acids (Ta^MGA3put^). These SNPs could be confirmed when the corresponding region was PCR amplified from MGA3 total DNA and sequenced in several parallels (data not shown). Thus, these SNPs were not simply due to previous sequencing errors.

### Enzyme assays in crude extracts demonstrated Ta activities for both strains

In order to test if Ta activity can be detected in strains *B. methanolicus* PB1 and MGA3, crude extracts were prepared from cells growing in defined methanol media. Surprisingly, both strains displayed Ta activities although MGA3 crude extracts contained significantly lower Ta activities (0.11 ± 0.02 and 0.24 ± 0.03 U/mg for MGA3 and PB1, respectively; Fig. 2b). We also tested just the assay mixture without added crude extracts or with crude cell extracts of a Ta negative *C. glutamicum* mutant ∆*tal* in order to exclude any background activity of the assay mixture*.* Ta activity was not detected when assaying just the assay mixture without added crude extracts or with crude cell extracts of a Ta negative *C. glutamicum* mutant ∆*tal*. A possible explanation for the observed Ta activity in *B. methanolicus* PB1 and MGA3 could be the presence of potential bifunctional enzymes with transaldolase activity, such as glucose-6-phosphate isomerase/transaldolase described for *Gluconobacter oxydans* [64]. However, a BLAST search with the nucleotide and amino acid sequences of Ta from *B. methanolicus* PB1 and sequences of other Ta variants against the MGA genome sequence did not reveal any putative Ta genes or proteins. In addition, also enzymes like fructose-6-phosphate aldolase, that might use the substrates of our Ta enzyme assay, were not found to be encoded in the MGA3 genome sequence.

### Construction of mutant ta^MGA3^ genes expressing full-length Ta proteins

In order to test if the Ta^MGA3^ sequence can be corrected to recreate a full-length TA, the ATA was changed to ATG to generate a translational start codon and the downstream T (indicated in pink in Fig. 3) was deleted to correct for a reading-frame shift. The corresponding protein was named Ta^MGA3rec^. As alternative, full-length Ta^MGA3^ proteins based on using the GTG as start codon (Ta^MGA3rec^) were constructed (named M1 to M6; Fig. 3). These variants differ with regard to the change of the TAA stop codon to different sense codons (Fig. 3).

### Heterologous expression of different MGA3 ta variants in *E. coli*

To test for Ta activity in crude cell extracts of *E. coli* DH5α*,* genes for the Ta variants M1-M6 and *ta*^*MGA3rec*^ (Fig. 3) were expressed. As suggested based on the bioinformatics analysis, Ta^MGA3*rec*^ showed Ta activity, but Ta^MGA3^ did not. This is likely due to the fact that Ta^MGA3^ lacks 80 amino acids that are present in the N-terminus of Ta^PB1^ and Ta^MGA3*rec*^. Recreation of functional Ta proteins was successful since Ta^MGA3rec^ and all six M1-M6 variants were found to be catalytically active (data not shown).

### Overexpression and purification of the Ta^MGA3rec^ and Ta^PB1^ proteins

The coding sequences for Ta^PB1^ and Ta^MGA3rec^ were cloned into the vector pET28b for production of the corresponding enzymes with a C-terminal His-tag in *E. coli* BL21 (DE3). Recombinant protein production was induced by the addition of IPTG to exponentially growing cell cultures. Stationary growth phase cells were harvested, crude extracts were prepared, and the proteins were purified by Ni-NTA chromatography. A buffer exchange to 50 mM Tris–HCl (pH 7.8) was performed and average concentrations of about 2.5 mg/mL were obtained. A total protein amount of about 7.5 mg was obtained per purification from 500 mL of culture broth. To determine the oligomeric state of the purified enzymes gel filtration and Ta activity assays of the eluted fractions were performed. The enzymes are active as homodimers with a molecular weight of 54 kDa since they eluted in a single fraction corresponding to that molecular weight. In the next steps we chose to focus on the biochemical characterization of wild-type Ta^PB1^, whereas Ta^MGA3rec^ was not studied any further.

### Biochemical characterization of Ta^PB1^

#### Optimal conditions for Ta^PB1^ activity

A coupled spectrometric assay for measuring the formation of GAP from F6P and E4P (as described in Materials & Methods) was used to determine optimal assay conditions for the Ta^PB1^. The auxiliary enzymes TIM and G3PDH were added in excess and found not to be limiting under the different conditions. Ta^PB1^ showed a pH optimum of pH 7.8. The highest activity was measured in the range of 60 °C to 65 °C (data not shown). The physiological temperature of 50 °C was chosen for further experiments and for determination of kinetic parameters.

#### Kinetic parameters of Ta^PB1^

We chose the conversion of F6P and E4P to S7P and GAP to determine the kinetic parameters of Ta^PB1^ (Table 1). All assays were performed in 50 mM Tris-HCl, pH 7.8, at 50 °C. A K_M_ of 740 μM and V_max_ of 16.3 U/mg was determined for the substrate F6P. The catalytic efficiency for F6P of 9.98 s^− 1^ mM^− 1^ could be derived. The corresponding kinetic constants for substrate E4P were K_M_ 2,5 mM and V_max_ 8.9 U/mg.

**Table 1:** Biochemical properties of Ta^PB1^

Parameter		Ta^PB1^
Molecular weight		54 kDa
Optimal conditions		50 mM Tris-HCl, pH 7.8, 50 °C
Optimal pH		7.2–7.4
Optimal temperature		60 °C
Temperature stability		< 60 °C
**Kinetics**		
F6P	K_M_	0.74 mM
	V_max_	16.3 U/mg
	k_cat_	7.35 s^−1^
	k_cat_/K_M_	10 s^− 1^ mM^− 1^
E4P	K_M_	2.5 mM
	V_max_	8.9 U/mg
	k_cat_	4 s^−1^
	k_cat_/K_M_	1.6 s^− 1^ mM^− 1^

Values for K_M_ (mM), V_max_ (U/mg), and catalytic efficiency (k_cat_/K_M_ = s^− 1^ mM^− 1^) were determined for two independent protein purifications and mean values and arithmetric deviations from the mean are given

### Heterologous expression of ta^PB1^ and Ta^MGA3rec^ complemented Ta-deficiency in *C. glutamicum ∆tal*

**Fig. 4:**
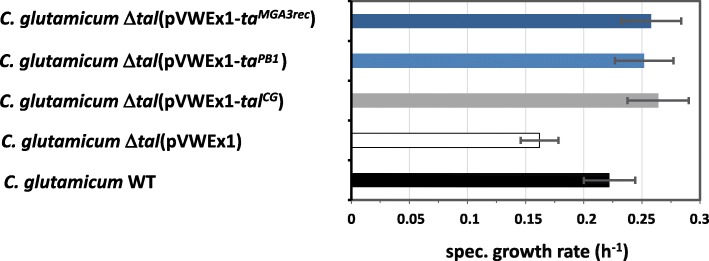
Genetic complementation of *C. glutamicum ∆tal* by expression of *ta*^*PB1*^, *ta*^*MGA3rec*^ and *tal*^*CG*^. The mean values and standard deviations for the specific growth rate of three replicates are shown for growth in CGXII minimal medium supplemented with 2% ribose

A genetic complementation experiment was performed to detect activity of Ta^PB1^ and Ta^MGA3rec^ in vivo*.* Since methods for gene deletion in *B. methanolicus* are lacking, a defined deletion mutant lacking Ta encoding gene *tal* was constructed in *C. glutamicum*. This bacterium possesses functional transaldolase (encoded by cg1776), which operates in the pentose phosphate pathway. Comparative growth analysis of *C. glutamicum ∆tal* and wild type revealed that *C. glutamicum* requires Ta for maximum growth rate in minimal medium with ribose as sole carbon source. *C. glutamicum ∆tal* could be complemented by expression of the endogenous *tal* from plasmid pVWEx1. Strain *C. glutamicum ∆tal* (pVWEx1-*tal*^*CG*^) grew with an even slightly higher specific growth rate than the wild type in CGXII minimal medium with 2% ribose. By contrast, the strain carrying the empty vector showed a reduced specific growth rate (Fig. 4). Thus, the deletion of *tal*, which is part of the *tkt-tal-zwf-opcA-pgl* operon in this bacterium, did not result in any secondary effects, such as polar effects on cotranscribed genes downstream of *tal*. In order to test if Ta from *B. methanolicus* PB1 is active in *C. glutamicum*, *ta*^*PB1*^ was heterologously expressed in the ∆*tal* strain. As this strain showed a similar specific growth rate as *C. glutamicum ∆tal* (pVWEx1-*tal*^*CG*^) (Fig. 4), expression of *ta*^*PB1*^ complemented *C. glutamicum* Δ*tal*. Since the same effect was observed for heterologous expression of *ta*^*MGA3rec*^, both Ta from *B. methanolicus* PB1 and the reconstructed version of the *B. methanolicus* MGA3 Ta protein are functionally active in vivo in *C. glutamicum*.

### Overexpression of *ta*^PB1^ in *B. methanolicus* MGA3

To test if overexpression of *ta*^PB1^ in *B. methanolicus* MGA3 would affect growth with methanol as sole carbon source, strains *B. methanolicus* MGA3(pHP13) and MGA3(pTH1mp-*ta*^PB1^) were constructed. However, growth in MeOH medium supplemented with different methanol concentrations was comparable. Thus, overexpression of *ta*^PB1^ in *B. methanolicus* MGA3 did not result in a growth advantage on methanol minimal media.

### Size exclusion chromatography, SDS-PAGE and MALDI-TOF analysis of MGA3 crude extracts

At this point, collected data and results from MGA3 were contradictory; i) the MGA3 genome has one Ta gene encoding a truncated and inactive protein, ii) MGA3 crude extracts display Ta activity, and iii) single point mutations can be introduced in the Ta gene restoring a full-length and catalytically active Ta protein. We therefore proceeded to investigate what could be the genetic and enzymatic cause of Ta activity in MGA3. First, Ta activity measured in a crude cell extract of *B. methanolicus* MGA3 (0.284 U) was used for size exclusion chromatography (gel filtration), in order to determine if Ta activity can be assigned to a particular fraction. Ta activity was confirmed for fractions 2 (0.174 U) and 8 (0.110 U) of a total of 18 fractions received, leading to 100% of the initial activity found in the crude cell extract. The two fractions showing Ta activity were separated using a SDS-PAGE and then every band was extracted from the gel and used for analysis by MALDI-TOF. However, peptide mass fingerprinting analysis did not allow to identify these proteins. This may be due to technical limitations in our laboratory, since, by contrast, the label-free quantitative proteomics analysis performed by [42] comparing growth of MGA3 with mannitol or methanol revealed that transaldolase I3EBM5 was not affected, whereas strong upregulation of fructose-1,6-bisphosphatase/sedoheptulose-1,7-bisphosphatase (GlpX) was observed [42]. Thus, there is evidence that MGA3 synthesizes transaldolase I3EBM5 or a variant sharing the MS detected peptides.

### Identification of a possible transcription start site upstream of the MGA3 ta gene

Since purified protein corresponding to the annotated Ta of *B. methanolicus* MGA3 (Ta^MGA3^) showed no activity when expressed in *E. coli,* however, crude extracts of MGA3 did show Ta activity, the available transcriptome data from RNA-sequencing [26] were used to search for a possible transcription start site (TSS) upstream of the start codon of our proposed Ta coding sequence *ta*^*MGA3put*^ (Fig. 3)*.* Indeed, a putative TSS preceded by a − 10 region (TTTCAA(T)) and a − 35 region (TTGAAA) were found. The − 35 region represents the consensus sequence [26], while the − 10 region shares only a low similarity with the *B. methanolicus* consensus sequence (TATAAT) [26].

### Proteome data indicating biosynthesis of functional Ta encoded by *ta*^*MGA3put*^

A search with the amino acid sequence of Ta^MGA3put^ against a database including proteome data from *B. methanolicus* MGA3 [42] identified peptides upstream of the annotated Ta (Müller, personal communication), which indicates transcription and translation of *ta*^*MGA3put*^ in MGA3. Taken together, our analysis of the available transcriptome and proteome data may indicate transcription of the proposed *ta*^*MGA3put*^*-*coding sequence, leading to the translation of a functional Ta protein responsible for the measured Ta activity in *B. methanolicus* MGA3. We did not try to determine kinetic constants of this putative Ta since the low activity present in the crude extracts was expected not to yield reliable data.

## Discussion

The focus of this study was a genetic and biochemical characterization of the transaldolase (Ta) from *B. methanolicus* [47] and to unravel its possible role in the RuMP cycle and thus methylotrophy. *B. methanolicus* wild-type strains MGA3 and PB1 display considerable physiological differences (growth rate, methanol consumption rate, respiration) in fed-batch methanol cultivations and these phenotypes are accompanied by certain genetic differences [23]. These two strains together therefore represent a valuable model system to investigate and understand methylotrophy, although MGA3 has been and still is the host for all metabolic engineering research of this organism. Both MGA3 and PB1 have one chromosomal putative Ta gene, and bioinformatics analysis presented here, indicated that the MGA3 gene has acquired point mutations in the 5′-region leading to a truncated gene product with a defective active site. Recombinant expression of the truncated protein in *E. coli* confirmed that it displays no catalytic activity. PB1 on the other hand encodes a full-length Ta gene and this gene was shown to express Ta activity when tested both in vitro and in vivo. Surprisingly, Ta activity was still measured in cell free extracts of both MGA3 and PB1. Totally seven genetically engineered versions of the MGA3 Ta gene were then constructed with different point mutations repairing the full-length version of the gene, and all seven gene products displayed Ta activity in vitro. Attempts to understand and explain this discrepancy in MGA3 as well as the biological role of Ta in *B. methanolicus* is further discussed below. Primary sequence alignments indicated that the *B. methanolicus* Ta^PB1^ protein belongs to the subfamily IV of Tas, similar as the Ta from *Bacillus subtilis.* Other well-characterized Ta proteins, such as TalB from *E. coli* and Ta from *C. glutamicum* belong to subfamily I and III, respectively.

Since the Ta^MGA3^ protein displayed no catalytic activity, the full-length and catalytically active Ta^PB1^ protein was used as *B. methanolicus* model protein for further purification and biochemical characterization in vitro. The kinetic constants for Ta^PB1^were calculated based on enzyme assays at the *B. methanolicus* relevant physiological conditions of 50 °C and pH 7.8. The reported kinetic constants for Ta of *E. coli, B. subtilis,* and *C. glutamicum* have been determined at 30 °C and pH 8.5 [51]. The measured K_M_ and V_max_ for substrates F6P (0.74 mM and 16 U/mg) and E4P (2.5 mM and 8.9 U/mg) of Ta^PB1^ are in the same range as those reported determined for the *B. subtilis* Ta (1.4 mM and 28 U/mg; 1.2 mM and 19 U/mg, respectively) [56]. While the K_M_ for F6P of TalB from *E. coli* (1.2 mM) and of Ta from *C. glutamicum* (1.3 mM) are in the same range as for Ta^PB1^, the V_max_ values of the latter enzymes are slightly higher (60 U/mg and 110 U/mg, respectively) [51, 60]. For the substrate E4P the K_M_ of TalB from *E. coli* (0.09 mM) and of Ta from *C. glutamicum* (0.7 mM) are lower, whereas their V_max_ values areslightly higher (80 U/mg and 84 U/mg, respectively) [51, 60], compared to Ta^PB1^.

Furthermore, the purified Ta^PB1^was found to have a temperature optimum of 60 °C in vitro, which is high compared to both TalB from *E. coli* (40 °C) [60] and Ta from *C. glutamicum* (40 °C) [51]. This was expected since *B. methanolicus* is a thermophilic bacterium with an optimal growth temperature of 50 °C to 55 °C [5, 53]. Further, TalB of *E. coli* and Ta of *C. glutamicum* are both reported to lose all catalytic activity at temperatures above 50 °C, whereas Ta of *B. subtilis* displayed increased activity when temperature was raised from 20 °C to 55 °C [51, 56, 60]. Thermostability is a common feature of Tas belonging to subfamily IV and V and not only restricted to thermophilic organisms [51]. It is proposed that a compact protein structure and a tight packing of the subunits causes this thermostability [66]. Summarized, Ta^PB1^ displays similar K_M_ and V_max_ values as Ta from *B. subtilis* and both proteins have temperature optima above 50 °C. Ta^PB1^ is however slightly different from TalB of *E. coli* and Ta of *C. glutamicum* with respect to K_M_ for substrate E4P, V_max_ values, and temperature optimum. Moreover, Ta^PB1^ was here demonstrated to form an active homodimer, whereas Ta of *B. subtilis* is reported to form a decamer (dimer of pentamers) [56], TalB of *E. coli* is a homodimer, and Ta of *C. glutamicum* is monomeric [51].

The active site is conserved among bacterial Ta proteins and it is also proposed that the catalytic mechanism is similar [51]. The important and highly conserved amino acid residue aspartate (Fig. 2a) acts as follows: The aspartate residue assists in the deprotonation of the C4 hydroxyl group of the enzyme-bound imine during the reaction catalyzed by Ta. This leads to the cleavage of the imine and releasing the first product, glyceraldehyde 3-phosphate (GAP). The resulting Schiff base intermediate is stabilized by resonance until the acceptor molecule is bound at the active site [30, 31, 50, 54, 55]. Since the truncated Ta^MGA3^ protein lacks this active site aspartate residue, this likely explains why no Ta activity was detected when expressed recombinant in *E. coli*.

Combined genomic and experimental analyses in MGA3 for identification of any other potential proteins with Ta activity, or that utilizes metabolites of the Ta assay, gave no results, and re-inspection of previous RNA-sequencing and proteome data indicated that a full-length Ta protein presumably using an upstream and in-frame GTG start codon is expressed in this strain. This is peculiar as the third codon in this putative *ta*^*MGA3put*^ gene would be the translational stop codon TAA, and the biological explanation to this remains unknown. Plausible explanation could be that this putative gene is subject to rare phenomena such as translational recoding which refers to alternative “translational decoding” [19, 58]. There are three different classes of such translational recoding described in nature: i) programmed-frame-shifting, ii) translational bypassing, and iii) translational redefinition of codons [7, 20]. Recoding is caused by special signals on the mRNA and is characterized by an unchanged genetic code as well as the synthesis of more than one protein from one mRNA [8]. In case of translational bypassing the ribosome stops translation at a certain point, skips some nucleotides, and then continues translation downstream [10]. For example, in *Bacillus firmus* two open reading frames have been found that are separated by a UGA stop codon and might be subject to read through [28]. In theory, the general mechanisms of translational bypassing or stop codon read through might serve as an explanation for the translation of a full-length and active Ta protein in MGA3. However, this remains to be experimentally tested and this was not within the scope of this study.

It has previously been shown that *B. methanolicus* uses the SBPase variant of the RuMP cycle [61] and three separate metabolomics studies have identified S7P in methanol grown MGA3 cells [13, 34, 43]. Our results presented here may indicate that *B. methanolicus* also possesses an active Ta variant of the RuMP cycle for regeneration of the formaldehyde acceptor Ru5P. The metabolomics analysis by [34] revealed similar labeling kinetics of SBP and S7P, which may indicate that the SBPase variant of the RuMP cycle is dominant in MGA3 grown in ^13^C-MeOH. Data from metabolic flux analysis in MGA3 have indicated that both RuMP variants are used in parallel (Carnicer, personal communication), and at the same time Ta expression is not upregulated during growth on methanol, neither on the transcriptome nor the proteome level [23, 42]. It has been shown that plants can use Ta besides SBPases [48]; however, to date no bacterium has been reported that uses the Ta and SBPase variant of the RuMP pathway in parallel.

## Conclusions

Evidence for an active transaldolase operating in *B. methanolicus* is presented based on biochemical characterization of the enzyme purified from recombinant *E. coli*. While it is well established that *B. methanolicus* can use the SBPase variant of the RuMP cycle this study indicates that *B. methanolicus* possesses Ta activity and may also operate the Ta variant of this cycle.

## Methods

### Microorganisms and cultivation conditions

*Bacillus methanolicus* strains were grown at 50 °C in the following media. SOBsuc medium is SOB medium (Difco) supplemented with 0.25 M sucrose. Methanol growth of *B. methanolicus* was performed in MeOH_200_ medium, containing salt buffer, 1 mM MgSO_4_, vitamins, trace metals, 0.025% yeast extract, and 200 mM methanol [29, 53]. The medium pH was adjusted to 7.2 unless stated otherwise. Bacterial growth was performed in shake flasks (500 mL) in 100 mL medium at 200 rpm and monitored by measuring the absorbance at 600 nm (A_600_). Chloramphenicol (5 μg/mL) was added to the medium when appropriate. The inoculation of the precultures for all growth experiments with *B. methanolicus* strains was performed with frozen ampoules of *B. methanolicus* as a starter culture. Ampoules of *B. methanolicus* cells were prepared from exponentially growing cultures (A_600_ of 1.0 to 1.5) and stored at − 80 °C in 15% (vol/vol) glycerol. Transformation of *B. methanolicus* MGA3 was performed by electroporation as described previously [29]. The *E. coli* strain DH5α was used as a standard cloning host. Recombinant cells were grown in lysogeny broth (LB) medium at 37 °C supplemented with kanamycin (25 μg/mL), spectinomycin (100 μg/mL), chloramphenicol (15 μg/mL), and 1 mM isopropyl-β-d-thiogalactopyranoside (IPTG) when appropriate and standard recombinant DNA procedures were performed as described elsewhere [49]. *C. glutamicum* cells were grown in lysogeny broth (LB) medium or CgXII minimal medium [32] and incubated at 30 °C. Recombinant protein production was carried out using *E. coli* BL21 (DE3) as host. Bacterial strains and plasmids used in this work are listed in Table 2 and oligonucleotides for polymerase chain reaction (PCR) and cloning are listed in Table 3.

**Table 2:** List of bacterial strains and plasmids used in this study

Strain, plasmid	Function and relevant characteristics	References
***B. methanolicus*strains**		
MGA3	Wild type strain, ATCC 53907	[53]
PB1	Wild type strain, NCIMBI 13113	[53]
***E. coli*strains**		
DH5α	General cloning host. F^−^*thi-1 endA1 hsdR17(r*^*−*^ m^−^) *supE44* ΔlacU169 (^−^80lacZΔM15) *recA1 gyrA96 relA1*	Bethesda Research Laboratories
BL21	protein expression host. *ompT hsdSB (rB*^*−*^ mB^_^) *gal dcm* (DE3)	Novagen
***C. glutamicum*strains**		
ATCC 13032	WT strain, auxotrophic for biotin	[1]
*Δtal*	In-frame deletion of the *tal* gene of WT	This study
**Plasmids**		
pVWEx1	Km^R^; *C. glutamicum*-*E. coli* shuttle vector (*P*_*tac*_*lacI*^*q*^*oriV*_*C.g*_*oriV*_*E.c*_)	[46]
pVWEx1-*ta*^*PB1*^	derived from pVWEx1, for regulated expression of *tal* of *B. methanolicus* PB1	This study
pVWEx1-*ta*^*MGA3rec*^	derived from pVWEx1, for regulated expression of modified *tal* of *B. methanolicus* MGA3	This study
pVWEx1-*tal*^*CG*^	derived from pVWEx1, for regulated expression of *tal* (cg1776) of *C. glutamicum* ATCC 13032	This study
pHP13	*B. subtilis*-*E coli* shuttle vector; Clm^R^	[22]
pTH1mp-*ta*^*PB1*^	pHP13 derivate with *tal* of *B. methanolicus* PB1 under control of *mdh* promoter	This study
pET28b	Kan^R^; T7*lac*; vector for his-tagged protein overproduction	(Novagen)
pET28b-*ta*^*PB1*^	purification of his-tagged *B. methanolicus* PB1 Ta from *E. coli* BL21(DE3)	This study
pET28b-*ta*^*MGA3rec*^	purification of his-tagged modified *B. methanolicus* MGA3 Ta from *E. coli* BL21(DE3)	This study
pET28b-*ta*^MGA3^_M1	derived from pET28b, for expression of variant M1 of *ta*^*MGA3*^ from *B. methanolicus* MGA3, see Table 3	This study
pET28b-*ta*^MGA3^_M2	derived from pET28b, for expression of variant M2 of *ta*^*MGA3*^ from *B. methanolicus* MGA3, see Table 3	This study
pET28b-*ta*^MGA3^_M3	derived from pET28b, for expression of variant M3 of *ta*^*MGA3*^ from *B. methanolicus* MGA3, see Table 3	This study
pET28b-*ta*^MGA3^_M4	derived from pET28b, for expression of variant M4 of *ta*^*MGA3*^ from *B. methanolicus* MGA3, see Table 3	This study
pET28b-*ta*^MGA3^_M5	derived from pET28b, for expression of variant M5 of *ta*^*MGA3*^ from *B. methanolicus* MGA3, see Table 3	This study
pET28b-*ta*^MGA3^_M6	derived from pET28b, for expression of variant M6 of *ta*^*MGA3*^ from *B. methanolicus* MGA3, see Table 3	This study
pET28b-*ta*^*MGA3*^	derived from pET28b, for expression of variant of the annotated *ta*^*MGA3*^ from *B. methanolicus* MGA3, see Table 3	
pK19mobsacB	Kan^R^; vector for gene deletions (RP4 *mob*; *sacB B. subtilis*; *lacZ;* OriV_E.c._)	[52]
pK19mobsacB-Δ*tal*	derived from pK19mobsacB for in-frame deletion of *C. glutamicum tal*	This study

Abbreviations: Spe^R^, spectinomycin resistance; Cm^R^, chloramphenicol resistance; Kan^R^ kanamycin resistance

**Table 3:** List of oligonucleotides used in this study

Name	Sequence (5–3′)
pET28b_Fw	GACTCACTATAGGGGAATTGTGAGCG
pET28b_Rv	AGATCCGGCTGCTAACAAAGCCCGA
pVWEx1_fw	CACTCCCGTTCTGGATAATG
pVWEx1_rv	GCTACGGCGTTTCACTTCTG
pTH1_fw	CTGCCCTTCCACCTTAACC
pTH1_rv	ATGTCACTAACCTGCCCCG
*ta*_MGA3rec-RBS-fw	GCGC**GGATCC**GAA*AGGAGG*CCCTTCAGATGGATGATTCAAAACAGTT
*ta*_MGA3rec-rv	GCGC**GGTACC**TTATTTCCCGCGTTTATTCC
*ta*_PB1-RBS-fw	GCGC**GGATCC**GAA*AGGAGG*CCCTTCAGATGATTCAAAACAGCATCCA
*ta*_PB1-rv	GCGC**GGTACC**TTATTGCCCGCGTTTTTTCC
*tal*_CG-RBS-fw	GCGC**GGATCC**GAA*AGGAGG*CCCTTCAGATGTCTCACATTGATGATCT
*tal*_CG-rv	GCGC**GGTACC**CTACTTCAGGCGAGCTTCCA
*ta*_PB1-TH-fw	GCGC**ACATGT**GATTCAAAACAGCATCCAACGAAAT
*ta*_PB1-TH-rv,	ATGC**GGTACC**TTATTGCCCGCGTTTTTTCC
*ta*_PB1_NcoI-fw	AGAG**CCATGG**ATGATTCAAAACAGCATCCA
*ta*_PB1_Xho-rv	AGAG**CTCGAG**TTGCCCGCGTTTTTTCCAATCTG
*ta*_MGA3rec_NcoI-fw	AGAG**CCATGG**ATGATTCAAAACAGTTTCCAACCAAATAAAG
*ta*_MGA3rec_Xho-rv	AGAG**CTCGAG**TTTCCCGCGTTTATTCCAGTC
*tal_*cg_del_A	GCGCGGATCCGGCTCCGGCTCCGAGGTTCA
*tal_*cg_del_B	*CCCATCCACTAAACTTAAACA*GAGCTGTGCAAGATCATCAA
*tal_*cg_del_C	*TGTTTAAGTTTAGTGGATGGG*CTTGAGTCCATGGAAGCTCG
*tal_*cg_del_D	GCGCGGATCCGCGGGTTTTGTCGATGCGCT
*tal_*cg_del_E	CTGCGTCCTGCAGATGCGAA
*tal_*cg_del_F	GGTCGATGCGGAACACAGAA
*ta*_MGA3,M1_BamHI-fw	CGC**GGATCC**GTGTCATATTTTAATACAGTT
*ta*_MGA3,M2_BamHI-fw	CGC**GGATCC**GTGTCAAAATTTAATACAGTT
*ta*_MGA3,M3_BamHI-fw	CGC**GGATCC**GTGTCATTATTTAATACAGTT
*ta*_MGA3,M4_BamHI-fw	CGC**GGATCC**GTGTCATCATTTAATACAGTT
*ta*_MGA3,M5_BamHI-fw	CGC**GGATCC**GTGTCACAATTTAATACAGTT
*ta*_MGA3,M6_BamHI-fw	CGC**GGATCC**GTGTCAGAATTTAATACAGTT
*ta*_MGA3,M_BamHI-rv	CCCC**GGATCC**TTATTTCCCGCGTTTATTCC
*ta*_MGA3_fw	
*ta*_MGA3_rv	

Restriction sites are highlighted in bold, linker sequences for crossover PCR and ribosomal binding sites are shown in italics, stop and start codons are underlined

The *C. glutamicum* wild type (WT) strain (ATCC 13032) and the derived Δ*tal* mutant, lacking transaldolase activity, were used for the heterologous expression of the Ta genes *ta*^*PB1*^ and *ta*^*MGA3rec*^ from *B. methanolicus* PB1 and MGA3, respectively. The vector pVWEx1 was used for IPTG-inducible expression of *ta*^*PB1*^. For growth experiments, *C. glutamicum* cells were harvested from cultures grown in LB medium overnight by centrifugation (4000 g for 10 min), washed in CgXII minimal medium, and used to inoculate fresh CgXII minimal medium. All growth experiments with *C. glutamicum* were carried out with 50 ml medium in 500 mL baffled shake flasks at 30 °C and 120 rpm. Growth was monitored by determination of the absorbance at 600 nm (A_600_) until the stationary phase was reached.

### Construction of *tal* (cg1776) deletion mutant in *C. glutamicum*

PCR products from chromosomal *C. glutamicum* DNA were obtained using primer pairs *tal*_cg_del_A; *tal*_cg_del_B and *tal*_cg_del_C; *tal*_cg_del_D. The resulting PCR products were used as template-DNA in a crossover PCR using primer pair *tal*_cg_del_A; *tal*_cg_del_D. The resulting PCR product with a shortened *tal* gene was then phosphorylated and ligated into the *Sma*I restricted vector pK19mobsacB [52]. Chromosomal deletion was performed as described elsewehere [16], and correct deletion was confirmed by sequencing using primers *tal*_cg_del_E; *tal*_cg_del_E. The constructed *C. glutamicum* Δ*tal* strain was used for the heterologous and homologous expression of different Ta variants.

### Vector constructions for heterologous expression of *ta*^PB1^ and *ta*^*MGA3rec*^ of *B. methanolicus* and homologous expression of *tal*^*CG*^ in *C. glutamicum*

The PCR products from *ta*^*PB1*^ and *ta*^*MGA3rec*^ were generated from genomic DNA of *B. methanolicus* PB1 and MGA3 by PCR, using the oligonucleotide primer pairs *ta*_PB1-RBS-fw; *ta*_PB1-rv and *ta*_MGA3rec-RBS-fw; *ta*_MGA3rec-rv (Table 3). The PCR product from *tal*^*CG*^ was generated from genomic DNA of *C. glutamicum* ATCC 13032 by PCR using the oligonucleotide primer pair *tal*_CG-RBS-fw; *tal*_CG-rv (Table 3). The amplified PCR products were digested with *BamH*I and *Kpn*I and then ligated with the *BamH*I and *Kpn*I restricted vector pVWEx1 [46]. The resulting vectors were named pVWEx1-*ta*^*PB1*^, pVWEx1-*ta*^*MGA3rec*^, and pVWEx1-*tal*^*CG*^. The vector pVWEx1 allows IPTG-inducible gene expression in *C. glutamicum* and *E. coli*. All resulting vector inserts were sequenced using the primer pair pVWEx1_fw; pVWEx1_rv to confirm their sequence integrity.

### Vector construction for heterologous expression of different *ta*^*MGA3*^ variants in *E. coli*

For the heterologous expression of the different *ta*^*MGA3put*^ variants M1-M6 in *E. coli* DH5α**,** the primers *ta*_MGA3,M1_BamHI-fw to *ta*_MGA3,M6_BamHI-fw; *ta*_MGA3,M_BamHI-rv (Table 3) were used for amplification by PCR from chromosomal DNA of *B. methanolicus* MGA3. The PCR product of the annotated *ta*^*MGA3*^ gene was generated using primers *ta*_MGA3_fw; *ta_*MGA3_rv. The resulting PCR products were digested with *BamH*I and ligated into the *BamH*I restricted vector pET28b (Table 3). Ta activity in crude cell extracts of recombinant *E. coli* DH5α strains was determined as described below.

### Vector construction for homologous overexpression of *ta*^*PB1*^ in *B. methanolicus* MGA3

The expression vector pTH1mp was used to allow methanol-inducible expression of the *B. methanolicus* PB1 Ta gene. This vector is analogous to the plasmid pHP13, in which the strong *mdh* promoter was cloned in-frame with the *mdh* RBS region to allow methanol-inducible expression in *B. methanolicus*. The DNA fragment of the *ta*^*PB1*^-coding region was amplified from DNA of *B. methanolicus* by the primer pair *ta*_PB1-TH-fw; *ta*_PB1-TH-rv (Table 3). The resulting PCR product was digested with *Pci*I/*Kpn*I and ligated with the *Pci*I/*Kpn*I digested vector pTH1mp. The resulting vector was named pTH1mp-*ta*^*PB1*^ and the correct insert was verified by sequencing using the primer pair pTH1_fw; pTH1_rv. Crude cell extracts were prepared based on the protocol described elsewhere [11]. The cells were inoculated from a glycerol stock and grown in MeOH_200_ medium overnight before they were transferred to fresh MeOH_200_ medium and grown to an A_600_ of 1.5 to 2.0. 40 mL of the cell culture was harvested by centrifugation (4.000 g, 30 min, 4 °C), washed in 50 mM potassium phosphate buffer (pH 7.8), and stored at − 20 °C. The cells were disrupted by sonication. Cell debris was removed by centrifugation (14.000 g, 60 min, 4 °C) and the supernatant was collected as crude extract. Ta activity was measured according to the conditions of the assay described below.

### Purification and molecular mass determination of Ta proteins

For protein production in *E. coli* BL21 (DE3) [63], *ta*^*PB1*^ and *ta*^*MGA3rec*^ were amplified by PCR using the primer pairs *ta*_PB1_NcoI-fw; *ta*_PB1_Xho-rv and *ta*_MGA3rec_NcoI-fw; *ta*_MGA3rec_Xho-rv (Table 3). After restriction with *Nco*I and *Xho*I the resulting PCR products were ligated into *Nco*I and *Xho*I restricted pET28b (Novagen, Madison, Wisconsin, USA), resulting in pET28b-*ta*^*PB1*^ and pET28b-*ta*^*MGA3rec*^. The pET28b vector allows the production of a C-terminal hexahistidine (His)-tagged Ta in *E. coli* BL21 (DE3). Protein production and purification were performed as described previously [38]. The enzyme was purified to homogeneity as verified by a 12% sodium dodecyl sulfate-polyacrylamide gel electrophoresis (SDS-PAGE) [37]. The protein concentration was measured according to the method of Bradford using the Sigma Bradford reagent with bovine serum albumin (BSA) as a standard. The dimeric structures of the Ta proteins were determined by gel filtration as described previously [38] using 1 mg Ta dissolved in 2 ml of 20 mM Tris-HCl, pH 7.8.

### Enzyme assays for the purified Ta proteins

To study the thermal stability of the Ta proteins, the assay mixture described below was prepared in 1.5 mL reaction tubes and incubated for up to 2 h at 30 °C to 80 °C. Samples were taken periodically and the residual enzyme activity was measured under standard conditions in a separate reaction mixture.

The Ta activity in the direction of S7P and GAP from E4P and F6P was determined. The standard reaction mixture (final volume 1 mL) contained 50 mM Tris-HCl buffer (pH 7.8), 0.25 mM nicotinamide adenine dinucleotide (NADH), triosephosphate isomerase (TIM), glycerol 3-phosphate dehydrogenase (G3PDH) and purified Ta protein which was preheated for 3 min at 50 °C. NADH (ε_340nm_ = 6.22 mM^− 1^ cm^− 1^) oxidation was followed at 340 nm on a Shimadzu UV1700 spectrophotometer. The reaction was initiated by the addition of E4P or F6P respectively (final concentration varied 0.05–10 mM).

### Tryptic digestion of proteins and mass spectrometry analysis

Protein samples displaying Ta activity were separated using a SDS-PAGE and the bands were excised from the gel and put in clean reaction tubes. The tubes were treated with an aqueous solution containing 60% (v/v) acetonitrile and 0,1% (v/v) trifluoroacetic acid (TFA) for removal of softener and dried overnight. The protein bands were treated two times with an aqueous solution containing 0.1 M ammonia carbonate and 30% (v/v) acetonitrile for 10 min each with soft shaking. The supernatant was removed and the fragments were dried in an Eppendorf SpeedVac. The dried fragments were treated with a trypsin solution (1 μL trypsin + 14 μL 10 mM ammonia carbonate, Promega) for 30 min at 21 °C. After addition of 20 μL 10 mM ammonia carbonate solution the samples were incubated for 12 h at 37 °C. The trypsin-digested samples were dried in the SpeedVac for 30 min. After the addition of 10 μL 50% (v/v) acetonitrile and 0.1% (v/v) TFA the samples were spotted onto a 800 μm Burker Anchor Chip, following the Burker Daltronics protocol. An Ultraflex matrix-assisted laser desorption/ionization time-of-flight mass spectrometer (MALDI-TOF-MS) (Bruker, Bremen, Germany) was used to obtain the corresponding peptide mass fingerprints, using the standard manufacturer’s parameters. The proteins were identified from a primary sequence database of *B. methanolicus* MGA3 using the Mascot (Matrix Science, London) search engine.

### Computational analysis

Sequence comparisons were carried out using protein sequences obtained from the NCBI database (http://www.ncbi.nlm.nih.gov) and using BLAST (*Basic Local Alignment Search Tool*) (http://blast.ncbi.nlm.nih.gov/Blast.cgi) [2].

## Data Availability

All data generated or analysed during this study are included in this published article. The datasets used and/or analysed during the current study are available from the corresponding author on reasonable request.

## Abbreviations

DHAP: Dihydroxyacetone phosphate
E4P: Erythrose 4-phosphate
Fba: Fructose-1,6-bisphosphate aldolase
Fba^C^: Chromosomally encoded fructose-1,6-bisphosphate aldolase
Fba^P^: Plasmid encoded fructose-1,6-bisphosphate aldolase
FBP: Fructose 1,6-bisphosphatase
GAP: Glyceraldehyde 3-phosphate
H6P: Hexulose 6-phosphate
Hps: 3-hexulose-6-phosphate synthase
Mdh: Methanol dehydrogenase
Pfk: Phosphofructokinase
Phi: 6-phospho-3-hexuloisomerase
Rpe: Ribulose-phosphate 3-epimerase
Rpi: Ribose-5-phosphate isomerase
Ru5P: Ribulose 5-phosphate
RuMP cycle: Ribulose monophosphate cycle
S7P: Sedoheptulose 7-phosphate
SBP: Sedoheptulose 1,7-bisphosphate
SBPase: Sedoheptulose-1,7-bisphosphatase
Ta: Transaldolase
Tkt: Transketolase
